# From histone acetylation to abundance: The role of GCN5-CAMTA2 interaction in wheat grain weight

**DOI:** 10.1093/plcell/koae257

**Published:** 2024-09-20

**Authors:** Regina Mencia

**Affiliations:** Assistant Features Editor, The Plant Cell, American Society of Plant Biologists; Instituto de Agrobiotecnología del Litoral (CONICET-UNL), Cátedra de Biología Celular y Molecular, Facultad de Bioquímica y Ciencias Biológicas, Universidad Nacional del Litoral, 3000 Santa Fe, Argentina

For genes to be expressed, transcription factors must bind to the promoters to initiate the process. Histone modifications play a critical role in controlling chromatin compaction and accessibility for transcription factors, thus tightly regulating the transcriptional program of cells. Histones can undergo various modifications, including the transfer of acetyl groups from acetyl-CoA to lysine residues by histone acetyltransferase (HAT) proteins. This acetylation reduces the positive charge on histone lysine residues, leading to chromatin relaxation, which allows transcription factors to easily access chromatin (Kumar et al. 2021).

*General Control Non-Depressible 5* (*GCN5*) encodes an HAT found in yeast, animals, and plants that acts on diverse histone substrates, such as H3K9, H3K14, H3K23, H3K27, H3K36, H2, and H4. In wheat (*Triticum aestivum*), GCN5 interacts with various transcription factors and is involved in regulating numerous developmental processes and stress responses, including resistance to powdery mildew and heat stress tolerance (Lin et al. 2022; Song et al. 2022). Although GCN5 has been determined to regulate multiple aspects of seed-related traits, such as high-molecular-weight glutenin content (Guo et al. 2015), its specific function in seed development has not been investigated to date. In new work, **Ruijie Zhang and colleagues (Zhang et al. 2024)** show that GCN5 interacts with the calmodulin-binding transcription factor CAMTA2 to regulate wheat grain size and weight.

Using CRISPR-Cas9, the authors generated *gcn5* mutants, which exhibited smaller grain width and weight. They found that this was due to an imbalance in starch content in seeds, thus pointing to a role of *GCN5* in regulating seed size. The authors used immunoprecipiation coupled with mass spectrometry to identify GCN5-interacting proteins that could explain the smaller seed size. Among the 62 identified proteins was the calmodulin-binding transcription factor CAMTA2. The specificity of this interaction was further validated using several techniques, including yeast 2-hybrid, in vitro pull-down assay, in vivo co-immunoprecipitation, and bimolecular fluorescence complementation. CRISPR-Cas9–generated *camta2* mutant plants exhibited smaller grain sizes, like the *gcn5* mutants, suggesting that CAMTA2 is involved in seed development.

To elucidate the downstream actions of the CAMTA2-GCN5 complex that could further explain the small seed phenotype, RNA-seq analysis was conducted on *camta2*, *gcn5*, and wild-type plants, focusing on genes involved in starch metabolism. Several genes, such as *Sus2-2A*, *Sus2-2B*, *SBEIc*, and *SBEIIb*, were found to be positively regulated by the complex. Electrophoretic mobility shift assay and chromatin immunoprecipitation followed by qPCR confirmed the direct binding of CAMTA2 to the *Sus2* and *SBEIc* promoters, along with GCN5. Additionally, the levels of H3K9ac and H3K14ac at these promoters were dependent on the presence of GCN5.

Interestingly, the authors observed that GCN5 and CAMTA2 could also act independently in the regulation of seed storage protein (SSP). While GCN5 positively regulates the expression of the glutenin (*Glu*) genes by directly binding to the promoter, CAMTA2 can also bind to *Glu* gene promoters and positively regulate their activity. However, in *camta2* mutants, GCN5 binding to *Glu* genes was not affected, suggesting that these 2 factors have independent roles.

Additionally, the authors analyzed a collection of 445 wheat germplasm accessions to search for single-nucleotide polymorphisms in the *CAMTA2-A* homolog. Three haplotypes were identified differing at 2 amino acid residues. Among them, the *CAMTA2-A^H3^* allele showed stronger binding affinity to the *Sus2* promoter and was strongly associated with accessions exhibiting larger grain sizes. Therefore, the *CAMTA2-A^H3^* allele represents a promising marker that might be exploited in breeding programs to develop wheat varieties with increased seed weight.

This work uncovers a molecular mechanism for the GCN5-CAMTA2 complex in regulating starch accumulation, alongside an independent role of GCN5 and CAMTA2 in controlling SSP accumulation (see Fig.). This research sheds light on the molecular processes underlying seed development and highlights the importance of histone acetylation in these processes.

**Figure.**
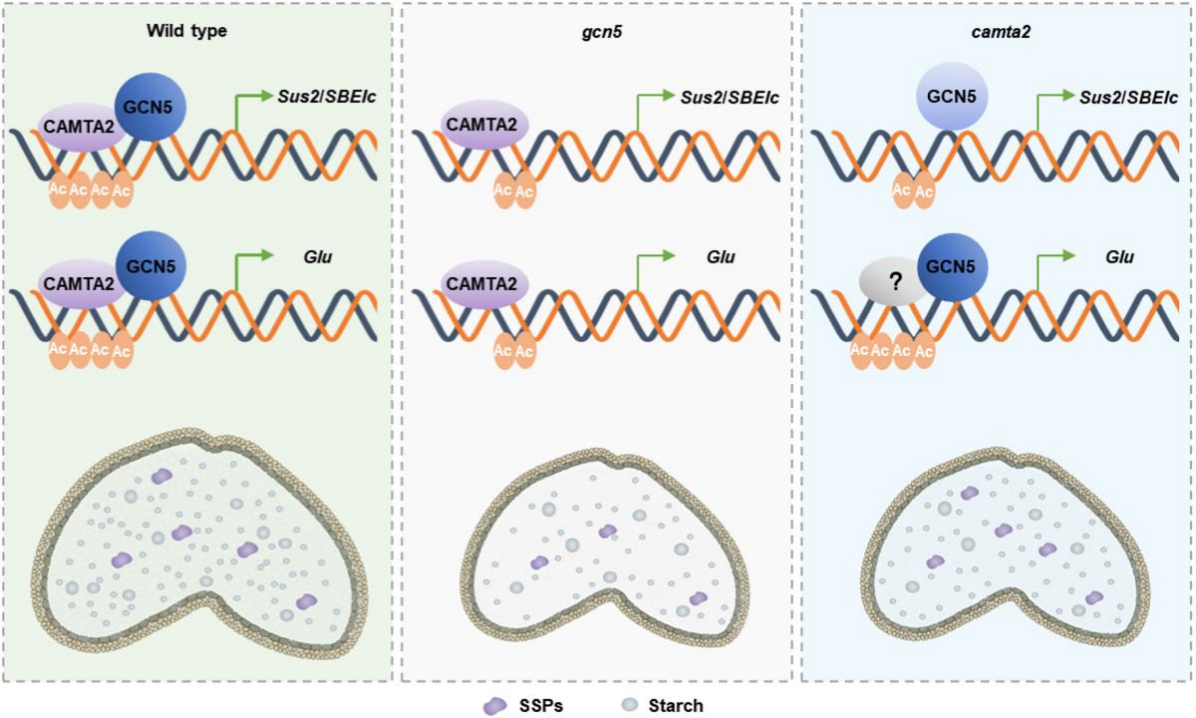
In wheat seeds, CAMTA2 binds *Sus2*, *SBEIc*, and *Glu* promoters, recruiting GCN5 to establish H3K9ac and H3K14ac, activating gene expression. Mutations in *GCN5* or *CAMTA2* reduce starch synthesis, resulting in smaller seeds. SSP accumulation is reduced in *GCN5* mutants but remains unchanged in *CAMTA2* mutants. Adapated from Zhang et al. (2024), Figure 8.

## Data Availability

No new data were generated or analysed in support of this article.

## Dive Curated Terms

The following phenotypic, genotypic, and functional terms are of significance to the work described in this paper:

histone CHEBI: CHEBI:15358
